# Five-Year Trend of Measles and Its Associated Factors in Pahang, Malaysia: A Population-Based Study

**DOI:** 10.3390/ijerph19138017

**Published:** 2022-06-30

**Authors:** Mohd Rujhan Hadfi Mat Daud, Nor Azwany Yaacob, Mohd Ismail Ibrahim, Wan Abdul Rahim Wan Muhammad

**Affiliations:** 1 Department of Community Medicine, School of Medical Sciences, Universiti Sains Malaysia, Kubang Kerian 16150, Kelantan, Malaysia; rujhan@student.usm.my (M.R.H.M.D.); ismaildr@usm.my (M.I.I.); 2 Pahang State Health Department, Ministry of Health Malaysia, Kuantan 25582, Pahang, Malaysia; drwanabdrahim@moh.gov.my

**Keywords:** measles, trend, incidence, associated factors

## Abstract

Measles is a disease that has resurfaced as a public health concern in Malaysia. Malaysia has had a Measles Elimination Program in place since 2004, but the incidence of measles in Pahang has not improved significantly. The purpose of this study was to describe the incidence and trend of measles in Pahang, as well as to identify the risk factors. The five-year population-based surveillance data of the entire Pahang state was extracted and analyzed for the trend and incidence of measles from January 2016 to December 2020. Multiple logistic regression was used to examine the relationship between measles and available sociodemographic data. A total of 2844 reported measles cases were investigated. Out of the measles cases reported, 7.41 percent were confirmed. According to the five-year fluctuating trend, the incidence rate ranges from 13.51 to 50.97 per 1,000,000 population. Confirmed measles was significantly associated with an indigenous background (AdjOR = 4.90, 95% CI: 1.74, 13.78), history of contact with measles cases (AdjOR = 14.03, 95% CI: 8.23, 23.90), and incomplete vaccination (AdjOR = 3.38, 95% CI: 2.28, 5.01). In conclusion, the incidence of measles in Pahang remains sporadic, owing to sporadic outbreaks. Vaccination is an important preventive measure that must reach out to the isolated populations such as indigenous people.

## 1. Introduction

Measles is an acute communicable viral disease. It is vaccine-preventable and characterized by fever, cough, runny nose, conjunctivitis, and pathognomonic enanthem of Koplik spots followed by the characteristic erythematous maculopapular rashes. Measles virus is a single-stranded RNA virus that is transmitted via respiratory droplets and by small suspended aerosol particles [1]. The incubation period is about 10 to 14 days, and the host is most infectious from four days before the onset of maculopapular rash up to four days after its appearance. The measles virus is highly infectious with an R naught (R_0_), or basic reproduction number, ranging from 12 to 18 [2]. Prior to the introduction of the measles vaccine in 1963, major epidemics of measles occurred every two to three years, with an estimated 2.6 million measles deaths each year. However, as the vaccine became widely available, a more than 84 percent decrease in measles deaths was observed between 2000 and 2016 [3]. In 2018, only 118 (61%) countries had achieved 90 percent measles vaccine first dose (MCV1) immunization coverage, and the global coverage of the measles-containing vaccine second dose (MCV2) was only 69 percent, which is still far from the target of 95 percent coverage [3].

Malaysia started the Measles Elimination Program in 2004 to reduce the incidence of measles and has implemented the Western Pacific Region Plan of Action for Measles Elimination and Field Guidelines for Measles Elimination. Measles-containing vaccines (MCV) have been in the national immunization schedule since 1982, initially given at the age of nine months. The two-dose trivalent measles–mumps–rubella (MMR) vaccine was introduced in 2004. Subsequently, in 2016, the vaccination schedule was shifted from twelve months old and seven years old to nine months old and twelve months old, respectively, as recommended by World Health Organization (WHO) [4]. In 2018, 43 percent of all countries were verified to have eliminated measles, with Iran and Sri Lanka being the most recent countries to do so [5]. The three main measles elimination strategies are to achieve and maintain 95 percent or greater MCV coverage with two doses, to conduct high-quality case-based measles surveillance, and to establish and sustain measles outbreak preparedness in order to provide rapid responses and appropriate case management [6]. Currently, measles notification and surveillance are carried out through two local online systems, namely, e-notification and SM2 e-measles. E-notification is an online system that notifies all suspected measles cases at all healthcare facilities, whereas SM2 e-measles is a system that standardizes reporting, investigations, and findings at the district, state, and national levels for measles control and prevention [4].

In 2004, Malaysia had proposed the elimination of measles by the year 2010. Despite good MCV coverage (96% for MCV1 and 99% for MCV2), the incident rate increased from 6.1 per million population in 2013 to 52.1 per million population in 2017. According to the Malaysian Ministry of Health (MOH), the number of measles cases in Malaysia increased exponentially from 195 in 2013 to 1934 in 2018, with the number of clusters increasing from 110 in 2013 to 133 in 2018. The target date for eradicating measles was later pushed back to 2025 [7]. Thus, trend analysis will be able to demonstrate disease control as a step toward elimination. The identification of factors that may contribute to the rise in measles incidence may facilitate future plans for disease prevention and control to meet the target of eliminating measles by 2025. The goals of this study are to describe the trend of measles in Pahang, as well as to determine the proportion of confirmed measles among notified measles cases in Pahang and the factors that contribute to it.

## 2. Materials and Methods

### 2.1. Study Setting

This was a cross-sectional study using population-based surveillance data from the Pahang state SM2 e-measles database from January 2016 to December 2020. Pahang is one of the states in Malaysia and is the third largest state, after Sarawak and Sabah, with a total land area of 35,965 km^2^. The total population of Pahang in 2019 was estimated to be 1.68 million. The major ethnic groups in Pahang are Malays, Chinese, Indian and Indigenous, with Malays constituting more than 70% of the total population [8]. Pahang is a state that is geographically dense with jungle and forest. Two-thirds of the state is covered with tropical rainforest, and the indigenous people mainly live in the remote areas within the forest [9].

Measles surveillance in Malaysia is based on a case classification system. A case is classified as either suspected or confirmed. A suspected case is any person diagnosed with measles by a clinician, and a confirmed case is a case that is laboratory confirmed or meets the clinical case definition and is epidemiologically linked to a laboratory-confirmed case [10]. In Malaysia, all suspected measles cases from government health care facilities or the private sector, by law, must be notified to the district health office. It is one of the diseases under mandatory notifiable diseases surveillance. Reporting or notifying infectious diseases is mandated by the Prevention and Control of Infectious Disease Act (PCID) 1988. All cases suspected of measles undergo laboratory testing via viral isolation or viral serology in line with the WHO recommendations for countries in the measles elimination phase.

All suspected cases of measles are notified electronically through SM2 e-measles. Cases are investigated within 48 h of notification. Laboratory testing is performed for confirmation, and case investigation carried out to determine any possible epidemiological link with other cases. This study used data from the SM2 e-measles database which contains both suspected and confirmed cases in the whole state of Pahang. Available data from SM2 e-measles are sociodemographic, medical history, diseases history, immunization history, and laboratory investigations. Pahang State Health Department is responsible for data collation of all measles notifications in Pahang.

### 2.2. Data Collection

All data from January 2016 to December 2020 were extracted from SM2 e-measles and saved using IBM SPSS IBM (Corp., Armonk, NY, USA) software version 27 with anonymous non-identifiable code. Two thousand eight hundred and sixty-six data were extracted for analysis. Data from non-Malaysian citizens or with at least one variable missing were excluded from analysis.

### 2.3. Operational Definitions

Suspected measles cases are cases that are seen and suspected as measles by a clinician via clinical assessment and in which they present with a fever, maculopapular rash and cough or coryza or conjunctivitis. A confirmed measles case is defined as laboratory confirmed, either by the presence of measles-specific IgM antibodies or the presence of measles virus in clinical samples using culture or molecular techniques, or as epidemiologically linked. A non-measles case is defined as any suspected case that is confirmed negative by laboratory findings, by either RT-PCR taken within 5 days of onset of rashes or IgM serology taken 4 days or more after the onset of rashes, or has equivocal IgM serology taken 4 days or more after the onset of rashes but a negative second sample taken 10 to 21 days post-rash, or has negative IgM serology taken less than 4 days after the onset of rashes and a negative second sample taken 6 days or more after first sample was taken. Measles vaccination status is categorized into complete vaccination, incomplete vaccination, and not vaccinated. Complete vaccination is defined as a case that has already received 2 doses of the measles vaccine. Incomplete vaccination is defined as a case that has already received 1 dose of the measles vaccine and not yet received the second dose of the measles vaccinate as per age. Not vaccinated is defined as a case that is not yet eligible for measles vaccination at the time of diagnosis in view of not yet having reached the age for the first dose of the MCV vaccine, or a case that did not receive any measles vaccination due to refusal or default. History of contact with measles cases is defined as any history of contact with measles cases within 21 days before the onset of rashes.

### 2.4. Statistical Analyses

The proportion of confirmed among notified measles cases was computed by the number of confirmed measles cases divided by the total number of notified measles cases from 2016 to 2020 and plotted in bar chart. The incidence rate of measles in Pahang was calculated by dividing the number of confirmed measles cases in a specific year by the mid-year population estimation for Pahang in the specific year for 1,000,000 population as estimated by the Department of Statistics Malaysia. The dependent variable of confirmed or non-measles was used to determine the association of measles infection with independent variables which were age, gender, ethnicity, urbanicity, history of contact with measles cases, history of visiting other countries, and measles vaccination status. Multiple logistic regression using the Forward Likelihood Ratio selection model was used. The adjusted Odds Ratio (AdjOR) with a 95% CI was used with the level of significance set at a *p*-value of less than 0.05.

## 3. Results

**Figure 1:**
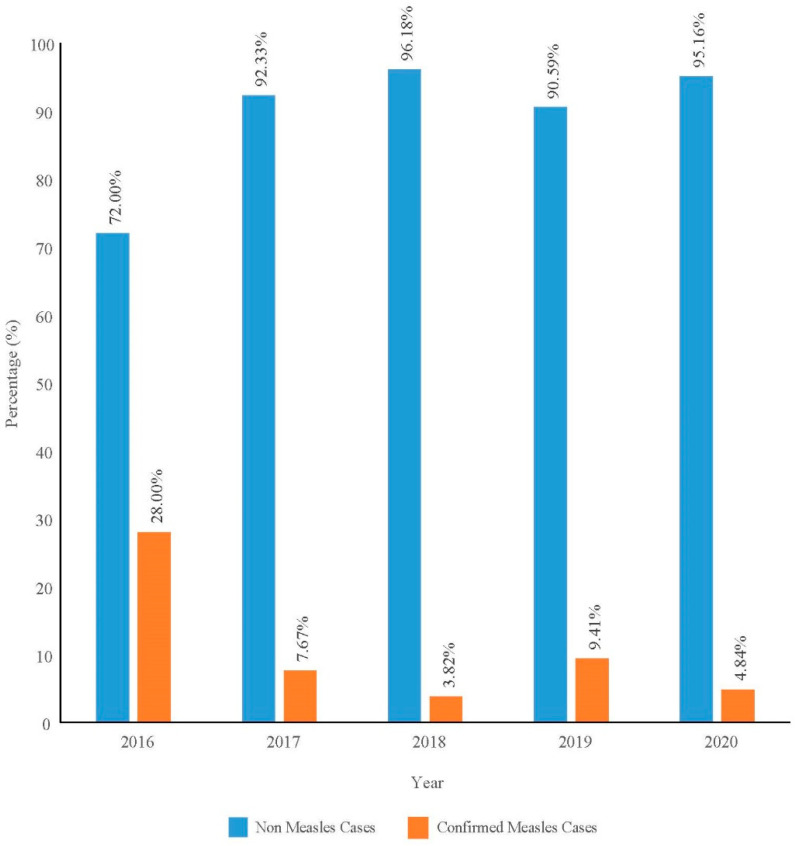
Proportion of confirmed measles among notified measles cases by year in Pahang from 2016 to 2020.

**Figure 2:**
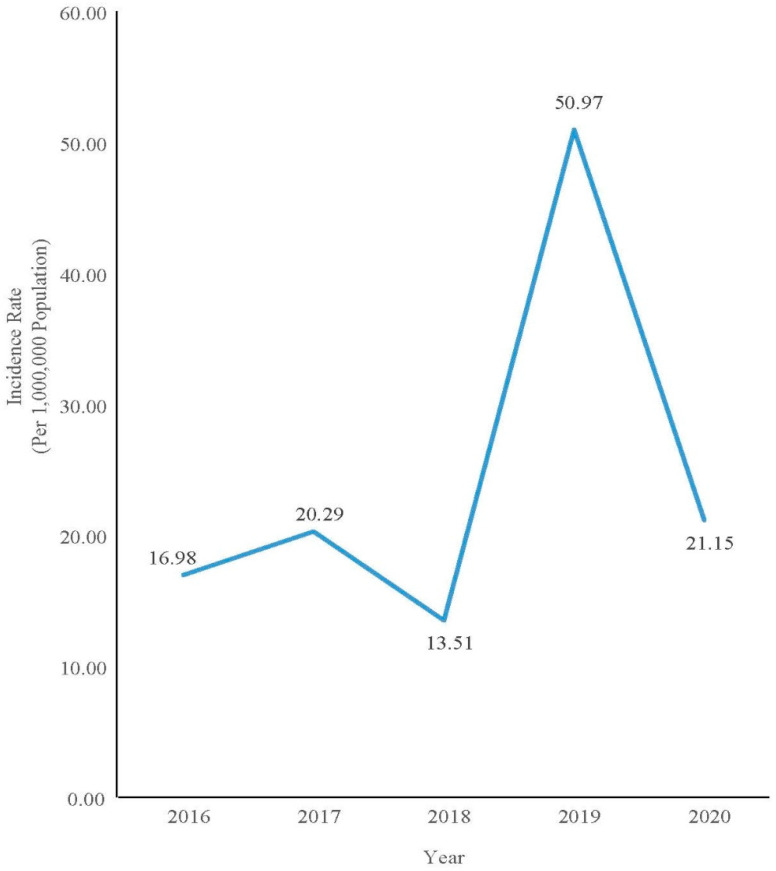
Incidence rate of confirmed measles cases in Pahang from 2016 to 2020.

A total of 2844 data points were analyzed from a total of 2866 notified cases. Nine were omitted due to insufficient data, and thirteen were non-Malaysian citizens. Only 7.41% (CI: 6.42, 8.34) of the cases were confirmed cases within the 5-year period. The proportion of confirmed cases by year ranged from 3.82% to 28.00% (Figure 1). The incidence rate, however, showed a fluctuating trend with a peak in 2019 (Figure 2).

A total of 2463 cases fulfilled the study definition of confirmed measles and non-confirmed measles from the 2844 cases of notified measles cases in this study. In total, 381 data points were excluded which were either diagnosed as vaccine associated measles or were non-valid data as the confirmatory lab samples were taken outside the appropriate sampling time frame for measles confirmatory testing.

The majority of the cases were less than 1 year old, thus not yet eligible for measles vaccination. Gender distribution was almost equal at a 1:1 ratio, and cases predominantly occurred among Malay ethnics. Most of them were reported in the Kuantan District and a large portion of cases were from urban areas. The majority had no history of contact with measles cases and had no history of travel to other countries in the past 21 days. For vaccination status, only 30 percent of the notified cases had complete measles vaccination (Table 1).

Multiple logistic regression analysis showed that ethnicity, history of contact with measles cases, and measles vaccination status are significantly associated with confirmed measles infection. Indigenous people have 4.90 times higher odds (95% CI: 1.74, 13.78, *p*-value = 0.003) of getting confirmed measles as compared with Chinese and Indian people. A person with history of contact with measles cases has 14.03 times (95% CI: 8.23, 23.90, *p*-value < 0.001) higher odds of getting confirmed measles as compared to those who do not have any history of contact with measles cases, and a person who has received incomplete vaccination has 3.38 times higher odds (95% CI: 2.28, 5.01, *p*-value < 0.001) of getting confirmed measles compared with those who have received complete vaccination (Table 2).

## 4. Discussion

The incidence of measles in Pahang varied over the course of five years. In 2019, the incidence rate increased to 50.97 per 1,000,000 population compared with 13.51 per 1,000,000 population in the previous year. The trend was different from the national trend, which shows that the national incidence rate was indeed reducing from 59.5 per 1,000,000 population in 2018 to 32.3 per 1,000,000 population in 2019 [11]. Measles incidence rates in Pahang deviated in 2019 due to outbreaks involving indigenous groups. In Pahang, two outbreaks involving indigenous people occurred in the districts of Jerantut and Lipis. The outbreak happened among the Bateq tribe. A similar outbreak had also occurred among the Bateq Tribe in Gua Musang, Kelantan, from May to July 2019. The outbreak occurred in Kuala Koh, Gua Musang, which is located at the border of Pahang. The Kuala Koh outbreak involved 110 confirmed measles cases and 11 deaths [11]. The outbreak in Pahang was thought to have originated from this nearby outbreak due to the nomad lifestyle of the Bateq tribe who moved around other neighboring states of Pahang, Kelantan, and Terengganu. One of the factors contributing to the outbreak was the low number vaccination recipients among the Bateq [12].

According to the Pahang State Health Department, three other outbreaks involving schools and pre-schools also occurred in Pahang in 2019. The high measles R_0_ of 12 to 18, as well as the proximity to areas such as schools and pre-schools, predisposed to more spread of measles infection in that area, resulting in a measles outbreak. This resulted in a total of five measles outbreaks in 2019 compared with only one measles outbreak in 2018. The increased occurrence of measles outbreaks may have contributed to an increase in the incidence rate of measles in Pahang that year. This is similar to a study conducted in Nigeria, which reported that the high frequency of outbreaks in Nigeria contributed to an increase in the incidence rate of measles [13].

In the current study, there was only a small percentage of confirmed measles cases among those who had been notified of a possible case of measles. The percentage of confirmed measles in this study was much lower than in previous studies, which ranged from 46.1 percent to 60.0 percent [3,14,15]. In Switzerland, clinically compatible measles other than laboratory-confirmed measles and epidemiologically linked measles were used to classify a case as a measles case [16]. A clinically compatible measles case is one in which there is a fever, rash, conjunctivitis, cough or coryza but no laboratory test and no epidemiologic link. Including clinically compatible measles cases among suspected measles cases may be one of the contributing factors to a higher percentage of cases classified as measles. Malaysia does not include clinically compatible measles cases in the measles definition because it is a tropical country with many diseases that can present with fever and rashes, such as dengue and chikungunya. However, notification of suspected measles includes clinically compatible measles detection methods.

The current study also found that the indigenous people are more likely to be infected with measles than other ethnicities. Peninsular Malaysia has a total indigenous population of 178,197 people, accounting for approximately 0.67 percent of Malaysia’s total population, with 67,506 of them being Pahang’s indigenous population. Pahang has 18 indigenous tribes, the majority of which live in Rompin, Pekan, and Lipis [17]. When compared to non-indigenous people, the indigenous population has lower vaccination coverage. During the measles outbreak in Kuala Koh, Gua Musang, indigenous MMR coverage for the first dose of measles-containing vaccine (MCV1) was only 61.5 percent [12]. Low vaccination coverage may be due to indigenous peoples’ difficult access to health services because of their lifestyle of living in remote villages and certain tribes being nomadic. Low vaccination coverage, poor diet, and poor hygiene all contribute to low immunity and a variety of indigenous health issues such as malnutrition, chronic diseases, and infectious diseases. These could be the reasons why indigenous people are more likely to contract measles. Because of their low immunity and ongoing health issues, they are at a higher risk of developing disease complications and are predisposed to a higher risk of death. This finding is consistent with the findings of an Australian study, which found that indigenous people were twice as likely as non-indigenous people to get measles, because the notification rate of measles among indigenous people is significantly higher than among non-indigenous people [18]. Another study in Australia found that the risk of getting complications from disease are 3 to 6 times higher and mortality are higher among the indigenous population compared with the non-indigenous [19]. In Malaysia, 11 people died as a result of measles complications in Kuala Koh and Gua Musang [12]. To serve the indigenous, special services such as a mobile team and a flying doctor squad have been organized. However, this service encountered logistical challenges such as poor road conditions, vehicle breakdowns and weather conditions. In 2019, only 41.6 percent of 173 planned flights were successfully conducted by flying doctor [11].

In Pahang, the odds of getting measles are 14.03 times higher if there is history of contact with measles cases. A case-control study in Ethiopia discovered that people who have had contact with measles within the last 21 days are 3.4 times more likely to get measles than those who have not had any contact with measles cases [20]. Similar findings were also observed in study in Indonesia and United Kingdom [21,22]. This finding could be explained by the fact that measles is spread by respiratory droplets, and close contacts were the most vulnerable group exposed to the droplets. The disease was extremely contagious, with 90% of non-immune people contracting it if exposed to an infective individual [23]. The infectivity period, which begins four days before the onset of the rash, increases the disease’s infectiousness because more people are exposed as they were unaware of the presence of the disease in an infected individual [24]. This finding supports the public health preventive measure of isolating children and not allowing them to attend school or nursery if they are suspected of having measles. Isolation reduces the possibility of contact and thus the possibility of measles transmission. The Malaysian government has provided quarantine leave for parents and caregivers of children with communicable diseases such as hand, foot and mouth disease, dengue fever, measles, chickenpox, diphtheria, and malaria [25]. This specific work leave eases the burden on the caretaker while also facilitating isolation.

As measles is one of the vaccine-preventable diseases, those who are under the age of one year and thus not yet received vaccination as well as those with incomplete vaccination are found to be at higher risk to be infected. This finding corresponds with an outbreak study in Merseyside, United Kingdom, in which incomplete vaccination was associated with measles infection [22]. One of the possible reasons is primary vaccine failure among the recipients of the MCV1 dose. Primary vaccine failure is defined as failure for a person to undergo adequate seroconversion following the first dose of the measles vaccine. Primary vaccine failure may occur due to an inadequate cold chain causing poor vaccine potency and an ineffective vaccine. Primary vaccine failure has been reported in Micronesia where it has caused an outbreak of measles [26]. A study in Selangor, Malaysia, found that 52.3% of health staff have poor knowledge of maintaining a vaccine cold chain in primary health care settings, which may contribute to ineffective vaccination [27]. A study on the optimal temperature of cold chains reported that 84.7% of the refrigerators were at suboptimal temperatures [28]. Another reason for vaccine ineffectiveness is due to giving a single-dose vaccination only. A single dose may provide only 85% protection if given at nine months old, whereas with two complete doses at appropriate intervals, measles vaccines are nearly 100% effective at protecting against measles [24].

Measles virus is highly contagious. Due to the nature of high contagiousness, near perfect vaccination coverage of 93 to 95% is needed to achieve herd immunity [29]. Herd immunity is needed to ensure the population is effectively protected against measles infection. In recent years, modified measles cases causing measles outbreaks have been reported in measles-eliminated countries such as Japan and Australia [30,31]. The diagnosis is difficult as the presentation of modified measles is atypical and is a milder form of the disease. It is commonly observed in cases with insufficient immunity that is contributed by primary or secondary vaccine failure [32]. Thus, booster doses may be required to overcome the vaccine failures and waning of measles immunity and further will maintain herd immunity.

### Limitation of the Study

Analysis based on surveillance data is limited by the completeness of the surveillance data on notifications and case investigations, particularly during large outbreaks. Comparisons for associated factors were also limited between confirmed and suspected cases thus limiting the generalizability to the general population.

## 5. Conclusions

Despite years of a measles vaccination program nationwide, measles cases in Pahang fluctuate due to the sporadic outbreaks. Unvaccinated by age and the nomadic lifestyle of the indigenous group demonstrates the importance of immunisation programs in prevention of measles as one of the public health vaccine-preventable disease concerns.

## Figures and Tables

**Table 1:** Sociodemographic and characteristics of notified measles cases in Pahang (*n* = 2463).

Variables	*n* (%)
**Age**	
<1 year old	1453 (59.0)
1 to 6 years old	833 (33.8)
7 to 12 years old	88 (3.6)
13 to 17 years old	28 (1.1)
≥18 years old	61 (2.5)
**Gender**	
Female	1169 (47.5)
Male	1294 (52.5)
**Ethnicity**	
Malays	2315 (94.0)
Chinese	69 (2.8)
Indian	3 (0.1)
Indigenous	76 (3.1)
**District**	
Kuantan	1151 (46.7)
Maran	285 (11.6)
Rompin	283 (11.5)
Temerloh	172 (7.0)
Jerantut	155 (6.3)
Bera	100 (4.1)
Raub	95 (3.9)
Bentong	78 (3.2)
Lipis	71 (2.9)
Pekan	57 (2.3)
Cameron Highlands	16 (0.6)
**Urbanicity**	
Rural	1018 (41.3)
Urban	1445 (58.7)
**History of contact with measles case**	
No	2390 (97.0)
Yes	73 (3.0)
**History of traveling to other countries in the past 21 days**	
No	2460 (99.9)
Yes	3 (0.1)
**M** **easles vaccination status**	
Complete vaccination	738 (30.0)
Incomplete vaccination	517 (21.0)
Not vaccinated	155 (6.3)
Not eligible for vaccination	1053 (42.7)

**Table 2:** Factors associated with confirmed measles among notified measles cases in Pahang (*n* = 2460).

Variables	Crude OR (95% CI)	*p*-Value *^a^*	Adjusted OR (95% CI)	*p*-Value *^b^*
**Gender**				
Female	1			
Male	0.91 (0.68, 1.22)	0.536		
**Ethnicity**				
Chinese and Indian	1		1	
Malay	0.87 (0.37, 2.04)	0.754	0.99 (0.41, 2.42)	0.983
Indigenous	6.42 (2.47, 16.71)	<0.001	4.90 (1.74, 13.78)	0.003
**Urbanicity**				
Rural	1			
Urban	1.10 (0.82, 1.48)	0.520		
**History of contact with measles cases**				
No	1		1	
Yes	14.53 (8.94, 23.61)	<0.001	14.03 (8.23, 23.90)	<0.001
**M** **easles v** **accination status**				
Complete vaccination	1		1	
Incomplete vaccination	3.23 (2.22, 4.70)	<0.001	3.38 (2.28, 5.01)	<0.001
Not vaccinated	0.89 (0.60, 1.30)	0.533	0.79 (0.52, 1.18)	0.246

*^a^* Simple Logistic Regression; *^b^* Multiple Logistic Regression; Constant = −2.954; Forward LR method applied; no multicollinearity and no interaction; Hosmer–Lemeshow test, *p*-value = 0.072; Classification Table 92.3% correctly classified; area under ROC curve 73.5%.

## Data Availability

The data are not publicly available due to privacy and confidentiality. Restrictions apply to the availability of data but are available for the authors with the permission of the organization.
